# The Probiotic Combination of *Lacticaseibacillus paracasei* JY062 and *Lactobacillus gasseri* JM1 Alleviates Gastrointestinal Motility Disorder via Improving Gut Microbiota

**DOI:** 10.3390/nu15040839

**Published:** 2023-02-07

**Authors:** Shasha Cheng, Hongxuan Li, Yixin Ding, Jiacheng Huo, Yaping Zheng, Yujun Jiang, Yu Zhang, Chaoxin Man

**Affiliations:** Key Lab of Dairy Science, Ministry of Education, College of Food Science, Northeast Agricultural University, Harbin 150030, China

**Keywords:** probiotics, gastrointestinal regulatory peptides, SCF/c-kit, gut microbiota, short-chain fatty acids

## Abstract

Probiotics have received wide attention as a potential way to alleviate gastrointestinal (GI) motility disorders. Herein, we investigated the effects of *Lacticaseibacillus paracasei* JY062, *Lactobacillus gasseri* JM1, and the probiotic combination at 5 × 10^9^ CFU/mL on mice induced by loperamide and explored the possible underlying mechanisms in GI motility disorder. After two weeks of probiotic intervention, the results indicated that the probiotic combination alleviated GI motility disorder better. It increased the secretion of excitatory GI regulators motilin, gastrin, and 5-hydroxytryptamine (5-HT) and decreased the secretion of the inhibitory GI regulators peptide YY and nitric oxide (NO), except vasoactive intestinal peptide. 5-HT and NO were related to the mRNA expression of 5-HT_4_ receptor and nitric oxide synthase, respectively. The intervention of probiotic combination also increased the number of interstitial cells of Cajal and the expression of SCF/c-kit protein. In addition, it also increased the abundance of beneficial bacteria (*Lactobacillus*, *Rikenellaceae*, and *Clostridiaceae_Clostridium)* and improved the contents of short-chain fatty acids in cecum contents of mice. In conclusion, the probiotic combination of *L. paracasei* JY062 and *L. gasseri* JM1 has the potential to alleviate GI motility disorders by balancing intestinal homeostasis.

## 1. Introduction

Gastrointestinal (GI) motility disorder is one of the main mechanisms triggering functional constipation and functional dyspepsia. Statistics showed that functional constipation has a prevalence of about 14% in adults, and 25–35% of patients with functional dyspepsia suffer from delayed gastric emptying and GI motility disorders [1,2]. Patients with GI motility disorder can present with varying degrees of abdominal distension, loss of appetite, nausea, halitosis, anxiety, and other symptoms [3]. Currently, drug therapy, such as laxatives and prokinetics, is still the main treatment method for GI motility disorders, with some limitations, including the single target of action, susceptibility to diarrhea, and high recurrence rate after drug discontinuation [4,5]. More new therapeutic options have been researched, such as probiotics, prebiotics, microbial metabolites, and natural ingredients from plants [6,7,8,9].

Most of the strains with probiotic functions are concentrated in *Lactobacillus* and *Bifidobacterium* [10], and the functions of probiotics have been well explained, such as regulating gut microbiota, alleviating lipid metabolism disorder, and improving inflammation [11,12,13]. Meanwhile, probiotics to alleviate GI motility disorders have received widespread attention, and they could avoid the limitations and side effects of drug therapy. More studies have demonstrated that *Lactobacillus plantarum* CQPC05, *Bifidobacterium lactis* TY-S01, and *Lactobacillus rhamnosus* strains play an essential role in alleviating GI motility disorder by influencing the production pathways of neurotransmitters, improving GI regulatory peptides, balancing gut microbiota, and regulating intestinal metabolites [6,14,15,16]. Additionally, different probiotic strains improve GI motility disorders in different alleviating ways. In addition, multiple strains were more advantageous than single strains and produced certain coordination effects to enhance their beneficial effects [17]. The probiotic compounds, including *Lactobacillus acidophilus* LA11-Onlly, *Lacticaseibacillus rhamnosus* LR22, *Limosilactobacillus reuteri* LE16, *Lactiplantibacillus plantarum* LP-Onlly, and *Bifidobacterium animalis* subsp. *lactis* BI516, could promote the colonization of specific strains and improve GI motility [18]. However, there are few studies on alleviating GI motility by the probiotic combination of *L. paracasei* and *L. gasseri*.

In this study, the regulatory effects of single probiotics and their combination on GI regulatory peptides, gut microbiota, and short-chain fatty acids (SCFAs) in mice with GI motility disorders were explored. Additionally, the mechanism of them to alleviate GI motility disorders was further investigated. This research could provide key insights for probiotics to alleviate GI motility disorders and provide a theoretical basis for this probiotic combination as a probiotic product and functional food.

## 2. Materials and Methods

### 2.1. Bacterial Strains and Culture Conditions

*L. paracasei* JY062 and *L. gasseri* JM1 were isolated from traditional fermented dairy products and healthy infant feces, respectively, and they were cultured in De Man, Rogosa, and Sharpe (MRS) broth (Qingdao Hope Bio-Technology Co., Ltd., Qingdao, China) at 37 °C under aerobic conditions for 20 h. Then, the cell pellets were collected by centrifugation at 5000× *g* for 5 min and resuspended in sterile (sterilized at 121 °C for 15 min and cooled to room temperature) phosphate buffer saline (PBS).

### 2.2. Mouse Model and Experimental Design

The male KM mice (*n* = 40, 6–7 weeks) were from Vital River Laboratory Animal Technology Co., Ltd., (Beijing, China). They were raised in cages with a room temperature of 22 ± 2 °C and a humidity of 55 ± 5% for 12 h of light/dark cycle. The experiment was designed and modified from the previous description [19]. All mice had free access to water and food for the first week to acclimatize to the environment. After seven days, the mice were divided into five groups (*n* = 8 per group), including the normal control group (NC), model group (M), *L. paracasei* JY062 group (JY), *L. gasseri* JM1 group (JM), and the combination group (MIX) (*L. paracasei* JY062: *L. gasseri* JM1 = 1:2). All groups of mice except the NC group were given 5 mg/(kg·d) body weight of loperamide by continuous gavage for 7 days. At this time, mice in the NC group were continuously gavaged with equal amounts of PBS. Then, mice of JY, JM and MIX groups were intragastrically administered *L. paracasei* JY062, *L. gasseri* JM1, and the combination with 5 × 10^9^ CFU/mL, at a dose of 10 mg/(kg·d) body weight for two weeks, respectively, whereas those of the NC and M groups were intragastrically administered PBS with an equal dose. Mice were anesthetized by intraperitoneal injection of ketamine and diazepam before sacrifice at the end of the experiment.

### 2.3. Physiological Indicators

Before being sacrificed, the mice were fasted for 24 h. Then, they were intragastrically administered 0.5 mL of ink and sacrificed 30 min later. The determination of the gastric emptying rate and small intestine propulsive rate was slightly modified from previous methods [20]. (1) Weighed the ink weight (*a*_1_) and total stomach weight (*a*_2_). Washed the stomach contents and weighed the stomach net weight after drying (*a*_3_). (2) Determined the distance from the pylorus to the front end of the ink (*L*_1_) and the distance from the pylorus to the ileocecal region (*L*_2_) with a ruler. (3) The fecal moisture content was determined by the low-temperature freeze-drying method. Weighed the plate weight (*b*_1_), the total weight of the plate and feces before freeze-drying (*b*_2_), and the total weight of the plate and feces after freeze-drying (*b*_3_). (4) Gastric antrum, small intestine, and colon samples were collected under sterile conditions. After soaking some tissues in 4% paraformaldehyde for 48 h, 5 mm-thick sections were used for hematoxylin and eosin (HE) staining to analyze the histological characteristics [21]. The formulas were as follows:

(1) $$\mathrm{Gastric}\;\mathrm{emptying}\;\mathrm{rate}\;(\%)=(1\;-\;\frac{a_{2}\;-\;a_{3}}{a_{1}})\;\times \;100\;$$

(2) $$\mathrm{Intestine}\;\mathrm{propulsive}\;\mathrm{rate}\;(\%)=\frac{L_{1}}{L_{2}}\;\times \;100\;$$

(3) $$\mathrm{Fecal}\;\mathrm{moisture}\;\mathrm{content}\;(\%)=\frac{b_{2}\;-\;b_{3}}{b_{2}\;-\;b_{1}}\;\times \;100\;$$

### 2.4. Determination of GI Regulatory Peptides and Neurotransmitters

The concentrations of motilin (MTL), gastrin (GAS), peptide YY (PYY), vasoactive intestinal peptide (VIP), and 5-hydroxytryptamine (5-HT) in serum were determined by commercially available ELISA kits (Nanjing Jiancheng Co., Ltd., Nanjing, China). The nitric oxide (NO) was determined by the nitric acid reductase biochemical kit (Beijing Solarbio Science & Technology Co., Ltd., Beijing, China).

### 2.5. Reverse Transcription (RT) and Quantitative Real-Time PCR (qPCR)

To investigate the expression of related genes (Aqp4, Aqp8, Thp1, 5-HT_4_R, SERT, c-kit, SCF, VIPR1, NOS), the total RNA of colon tissues was isolated by a Simple P Total RNA Extraction Kit (Bioer Technology Co., Ltd., Hangzhou, China). cDNA was synthesized by PrimeScript™ RT reagent Kit (TaKaRa Bio, Dalian, China). All procedures were performed by the manufacturers’ instructions. The mRNA levels were determined by the QuantStudio^®^ 3 Real-Time PCR system (Applied Biosystems, Foster City, CA, USA) with TB Green^®^ Premix Ex Taq™ II (TaKaRa Bio, Dalian, China). The relative expression of mRNA was calculated using the 2^−ΔΔCt^ method. The primers used in this study were listed in Table 1.

### 2.6. Immunohistochemistry and TUNEL Staining Analysis

The colon sections were dewaxed in xylene, and antigens were retrieved with citrate antigen retrieval solution, cooled to room temperature, blocked with goat serum for 1 h, and incubated with anti-c-kit antibody overnight at 4 °C, followed by incubation with biotinylated secondary antibody at room temperature for 1 h and horseradish enzyme-labeled avidin at 37 °C for 30 min. They were then stained by diaminobenzidine (DAB) and observed under a light microscope. The Mean Density (IOD/AREA) was determined with Image-pro Plus 6.0 (Media Cybernetics, Inc., Rockville, MD, USA).

The determination of apoptosis of interstitial cells of Cajal (ICC) was done by TUNEL staining analysis by a TUNEL apoptosis assay kit (Roche, Germany). According to the previous method, with a few modifications [22], paraffin sections of colonic tissues were treated with xylene and ethanol and washed with PBS. Proteinase K was added and incubated at 37 °C for 30 min, and 3% bovine serum albumin (BSA) was added and incubated at room temperature for 20 min. Then, it was incubated with 50 µL TUNEL staining solution at 37 °C for 2 h, incubated with c-kit primary antibody (1:100) overnight at 4 °C, and incubated with secondary antibody (1:400) in the dark at room temperature for 50 min. 4′,6-diamidino-2-phenylindole (DAPI) was added dropwise in the dark for 10 min and the slice was sealed with anti-fluorescence quenching sealer. The images were observed and acquired under a fluorescence microscope.

### 2.7. Western Blot (WB) Analysis

Determination of c-kit and SCF protein expression by WB analysis with modifications of previously described methods [23,24]. Specifically, frozen colonic tissue (100 mg) was added to protein lysate (1 mL) containing protease inhibitor cocktail (1:200) and homogenized thoroughly in an ice bath. The homogenate was centrifuged at 13,000× *g* for 5 min, and the supernatant was collected to determine the protein concentration using the bicinchoninic acid protein assay kit (ROCHE, Basel, Switzerland). Equal amounts of protein samples (40 µg) were separated by 10% SDS-PAGE (Bio-Rad, Hercules, CA, USA) and transferred to PVDF membranes (Millipore, Bedford, MA, USA). Membranes were blocked with 5% skim milk in Tris-buffered saline with 0.1% Tween-20 (TBST) at room temperature for 1 h. GAPDH was used as an internal reference. Membranes were incubated overnight with primary antibody (1:1000) at 4 °C. After washing in TBST, the secondary antibody (1:5000) was added and incubated at room temperature for 30 min, then the blots were washed with TBST and exposed to enhanced chemiluminescence-plus reagents (ECL) for chemiluminescence detection. The grayscale of the target bands was analyzed with Image-pro Plus 6.0 (Media Cybernetics, Inc., Rockville, MD, USA).

### 2.8. Gut Microbiota Sequencing and Analysis

Cecum contents from mice were collected to assess the changes in the composition of gut microbiota. The total DNA was extracted by using the DNA Extraction Kit (NucleoSpin, MN, Germany). PCR amplification of the V3–V4 region of the 16S rRNA gene was performed using primers (F: ACTCCTACGGGAGGCAGCAG, R: GGACTACHVGGGTWTCTAAT), as described previously [15]. DNA samples were sequenced by Illumina MiSeq. Bacterial diversity analysis of the 16S rRNA sequence data was conducted after sequencing.

### 2.9. Determination of SCFAs

Cecum contents from mice were homogenized and sonicated to make a suspension and centrifuged at 5000× rpm for 20 min. The extract (0.8 mL, 25 mg/L methyl tert-butyl ether stock solution) and 50% H_2_SO_4_ (0.1 mL) were added as internal standards to the supernatant (0.8 mL) and shaken for 10 min, sonicated for 10 min in ice bath water, centrifuged at 10,000× rpm for 15 min, and then transferred the supernatant to a fresh glass vial for GC-MS analysis according to Wang’s method [16].

### 2.10. Statistical Methods

The data were analyzed by one-way analysis of variance (ANOVA) using SPSS and Origin software. The results were expressed as mean ± standard deviation (Mean ± SD). Graphs were drawn using Excel software and GraphPad Prism 8.02.

## 3. Results

### 3.1. Effects on Physiological Indicators of Mice with GI Motility Disorder

**Figure 1:**
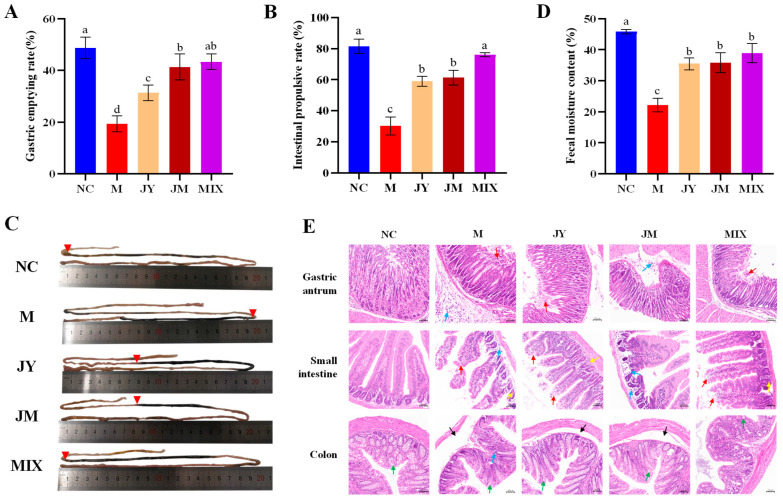
Alleviation of gastrointestinal (GI) motility disorder of probiotics: (**A**) change of gastric emptying rate in mice; (**B**) change of small intestinal propulsive rate in mice; (**C**) specifics of ink propulsion in the small intestine; (**D**) change of fecal moisture content in mice; (**E**) hematoxylin and eosin (HE) staining for gastric antrum, small intestine and colon sections (200×). Different letters (a, b, c and d) denote significant differences between different groups of mice (*p* < 0.05). NC stands for the normal control group, M for the model group, JY for the *L. paracasei* JY062 group, JM for the *L. gasseri* JM1 group, and MIX for the combination group. Red trangles in (**C**) stand the front end of the ink, and different color arrows in (**E**) denote eroded and shed mucosal epithelial cells (red arrows), inflammatory cells (blue arrows), submucosal edema (black arrows), Paneth cells (yellow arrows) and goblet cells (green arrows).

As shown in Figure 1A–C, the gastric emptying rate and intestinal propulsive rate in the M group were significantly lower than those in the NC group (*p* < 0.05), but the trend was reversed after the probiotic intervention, in which the MIX group could reach the NC group level. Specifically, the gastric emptying rate in the MIX group (43.3 ± 3.1%) was not significantly different from that in the JM group (41.3 ± 5.0%), but it was significantly higher than that in the JY group (31.3 ± 3.1%). Additinally, the intestinal propulsive rate in the MIX group (76.1 ± 1.2%) was significantly higher than that in the JY group (59.0 ± 3.2%) and the JM group (61.2 ± 4.8%). Simultaneously, fecal moisture content was increased after intervention, which significantly improved the lower level of the M group (*p* < 0.05) (Figure 1D). These results indicated that *L. paracasei* JY062 and *L. gasseri* JM1 could promote GI motility to some extent, and their combination was more effective.

The results of the HE staining (Figure 1E) showed that the local mucosal epithelial cells in the gastric antrum of the M group were eroded and shed (red arrows), and a large number of inflammatory cells (blue arrows) were infiltrated in the lower layer. In addition, the villi of the small intestine were poorly structured, significantly shorter in length, and had inflammatory cells (blue arrows). Additionally, inflammatory cells were also seen in the colonic mucosa, and submucosal edema was evident (black arrows). After the intervention of *L. paracasei* JY062 and *L. gasseri* JM1, the injury of the gastric antrum, intestine, and colon were relieved to different degrees, and the improvement effect was more significant in the MIX group, which was close to the NC group. Notably, the presence of Paneth cells (yellow arrows) and goblet cells (green arrows) in the small intestine and colon may be related to the stress response of the body against the external environment. These results indicated that the intervention of single and combination of *L. paracasei* JY062 and *L. gasseri* JM1 could alleviate the degree of injury.

### 3.2. Effects on GI Regulatory Peptides and Neurotransmitters of Mice with GI Motility Disorder

**Figure 2:**
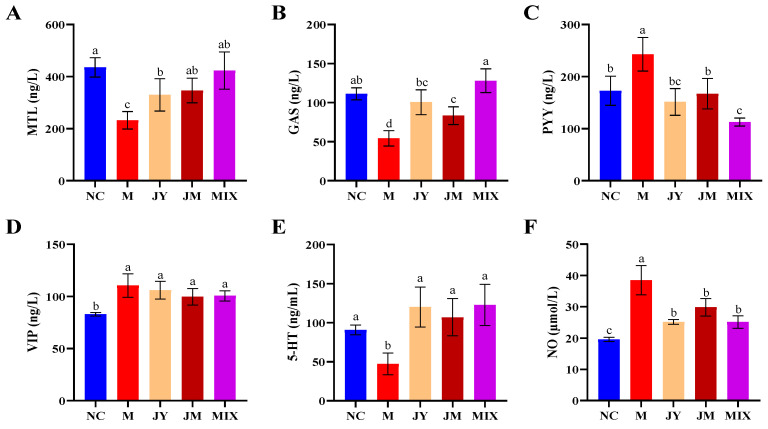
The contents of GI regulatory peptides and neurotransmitters in mice: (**A**) content of motilin (MTL); (**B**) content of gastrin (GAS); (**C**) content of peptide YY (PYY); (**D**) content of vasoactive intestinal peptide (VIP); (**E**) content of 5-hydroxytryptamine (5-HT); (**F**) content of nitric oxide (NO). Different letters (a, b, c and d) denote significant differences between different groups of mice (*p* < 0.05). NC stands for the normal control group, M for the model group, JY for the *L. paracasei* JY062 group, JM for the *L. gasseri* JM1 group, and MIX for the combination group.

The levels of GI regulatory peptides (MTL, GAS, PYY, VIP) and neurotransmitters (5-HT, NO) were determined. The results (Figure 2A,B) showed that the contents of MTL and GAS were improved after probiotic intervention, significantly higher than that of the M group (*p* < 0.05). The levels of MTL and GAS in the MIX group were 423.3 ± 71.67 ng/L and 128.1 ± 15.16 ng/L, respectively, and there was no significant difference compared with the NC group (*p* > 0.05). Previous studies have shown that PYY and VIP, the inhibitory regulators of GI hormones, could inhibit gastric acid secretion and GI motility [25]. Similarly, the PYY level in M group was significantly increased compared with the NC group (*p* < 0.05), and the trend was reversed after the intervention, especially in the MIX group (Figure 2C). However, except for the NC group, there was no significant difference in VIP levels between other groups (*p* > 0.05) (Figure 2D). The excitatory factor 5-HT and the inhibitory factor NO were essential in the regulation of GI motility. The results showed that, compared with the M group, the level of 5-HT was increased significantly after probiotic intervention (*p* < 0.05), whereas NO showed an opposite trend, and the degree was more obvious in MIX (Figure 2E,F). Thus, *L. paracasei* JY062, *L. gasseri* JM1, and, in particular, their combination, may regulate the level of GI regulatory peptides and neurotransmitters.

### 3.3. Effects on the Expression of Related Genes of Mice with GI Motility Disorder

**Figure 3:**
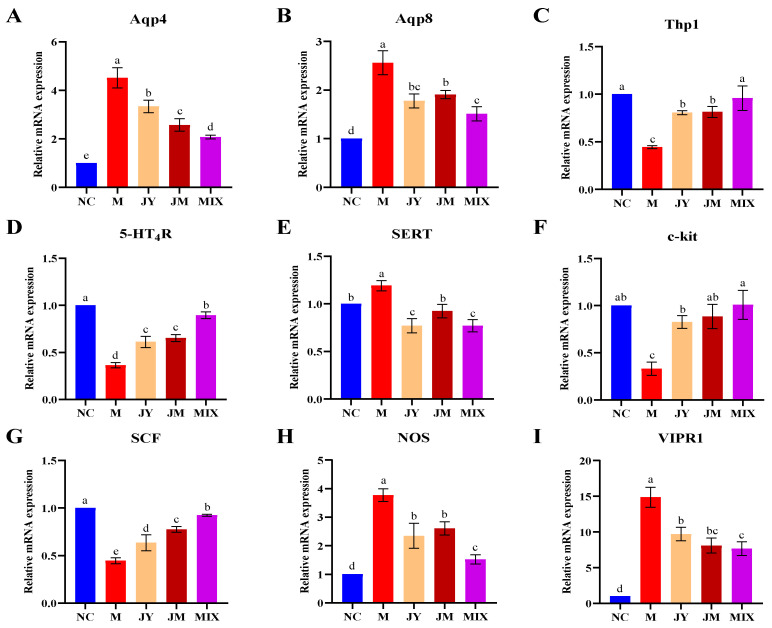
Regulatory effect of probiotics on related genes in mice: (**A**) mRNA relative expression of Aqp4; (**B**) mRNA relative expression of Aqp8; (**C**) mRNA relative expression of Thp1; (**D**) mRNA relative expression of 5-HT_4_R; (**E**) mRNA relative expression of SERT; (**F**) mRNA relative expression of c-kit; (**G**) mRNA relative expression of SCF; (**H**) mRNA relative expression of NOS; (**I**) mRNA relative expression of VIPR1. Different letters (a, b, c, d and e) denote significant differences between different groups of mice (*p* < 0.05). NC stands for the normal control group, M for the model group, JY for the *L. paracasei* JY062 group, JM for the *L. gasseri* JM1 group, and MIX for the combination group.

Aquaporin is the protein in cell membranes that selectively transports water molecules. The expression levels of Aqp4 and Aqp8 in the M group were significantly increased compared with the NC group (*p* < 0.05), whereas those were reversed after the intervention, especially in the MIX group (Figure 3A,B). The increased expression of Thp1 and 5-HT_4_R and the decreased expression of SERT indicated that they were improved by intervention, and Thp1 and 5-HT_4_R had more significant effects in the MIX group (Figure 3C–E). After the intervention, the expressions of c-kit and SCF were increased (Figure 3F,G), whereas those of NOS and VIPR1 were decreased (Figure 3H,I), indicating that the single and combination of *L. paracasei* JY062 and *L. gasseri* JM1 promoted the expression of pro-regulatory factors and inhibited the expression of anti-regulatory factors.

### 3.4. Effects on ICC, c-kit Protein and SCF Protein of Mice with GI Motility Disorder

**Figure 4:**
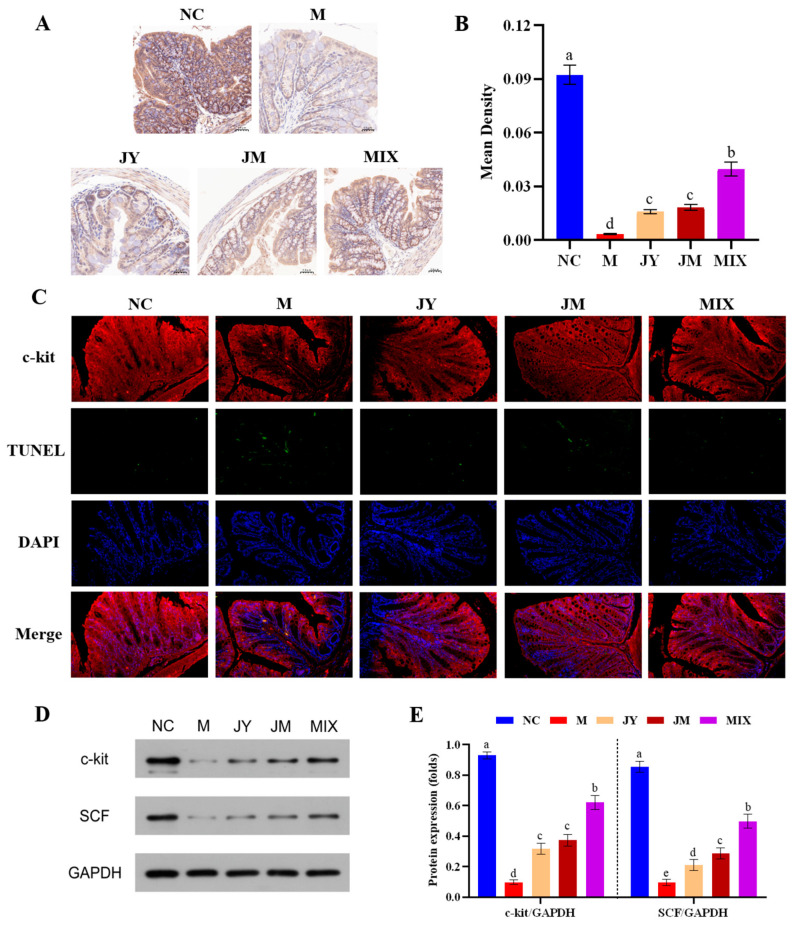
Description of interstitial cells of Cajal (ICC) and expression of c-kit and SCF protein in colon: (**A**) ICC distribution in colon (200×); (**B**) the Mean Density of c-kit in colon; (**C**) ICC apoptosis in colon (200×); (**D**) blot images of c-kit and SCF; (**E**) expression of c-kit and SCF protein. Different letters (a, b, c, d and e) denote significant differences between different groups of mice (*p* < 0.05). NC stands for the normal control group, M for the model group, JY for the *L. paracasei* JY062 group, JM for the *L. gasseri* JM1 group, and MIX for the combination group.

The c-kit is a transmembrane receptor that produces the primary signaling of ICC and is commonly used to label the amount of ICC [26]. The tissue section (200×) was shown in Figure 4A; the brown-yellow color part indicated the positive expression. The brown-yellow color part of the M group was seriously lighter compared with the NC group, whereas those of the JY and JM groups were increased, especially in the MIX group. The results of Mean Density (Figure 4B) showed that the level of positive expression in the MIX group was significantly higher than those in the JY and JM groups (*p* < 0.05), reflecting the distribution of ICC in the mice of each group and the relatively high number of ICC in the MIX group. In addition, as shown in Figure 4C, red fluorescence indicated positive labeling of c-kit and green fluorescence indicated cell apoptosis. The results showed an increase in the positive expression of c-kit after the probiotic intervention, consistent with the trend of immunohistochemical results. However, ICC apoptosis showed an opposite trend, with the highest level of ICC apoptosis in the M group. Additionally, the ICC apoptosis was alleviated after the probiotic intervention. Specifically, the level of ICC apoptosis in the MIX group was lower than those in JY and JM groups, which was close to the level of NC group. Simultaneously, the protein expression levels of both c-kit and SCF increased after probiotic intervention, with a significant increase in the MIX group (Figure 4D,E), similar to the results for gene expression of c-kit and SCF. It can be concluded that the expression of the c-kit and SCF protein was increased by the single and combination of *L. paracasei* JY062 and *L. gasseri* JM1, further promoting the amount of ICC increased after intervention.

### 3.5. Effects on Gut Microbiota of Mice with GI Motility Disorder

**Figure 5:**
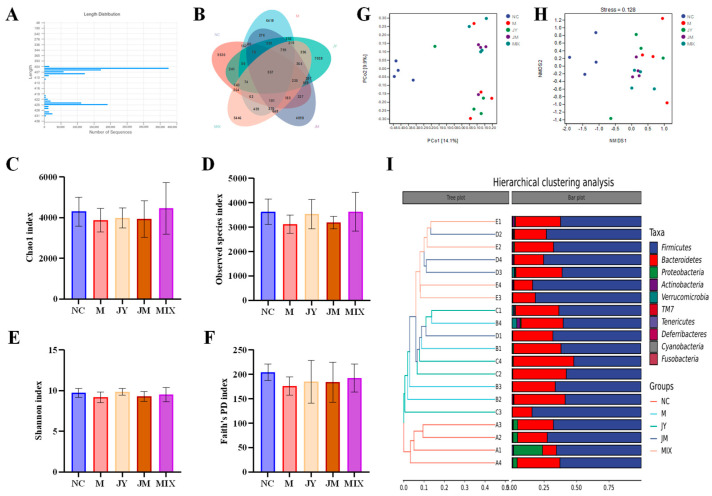
Alpha diversity index and Beta diversity analysis of gut microbiota: (**A**) sequence-length distribution; (**B**) ASV venn diagram; (**C**) Chao1 index; (**D**) Observed species index; (**E**) Shannon index; (**F**) Faith’s PD index; (**G**) principal coordinates analysis (PCoA); (**H**) nonmetric multidimensional scaling (NDMS) analysis; (**I**) hierarchical clustering analysis using the unweighted pair-group method with arithmetic means (UPGMA). (**A**) stands for the normal control group (NC), (**B**) for the model group (M), (**C**) for the *L. paracasei* JY062 group (JY), (**D**) for the *L. gasseri* JM1 group (JM), and (**E**) for the combination group (MIX).

The abundance and diversity of gut microbiota are essential in improving GI diseases. The effects of gut microbiota of mice with GI motility disorder were systematically studied and analyzed. The length distribution for amplicon sequence variants (ASVs) was determined (Figure 5A). Those in all samples would mainly range from 400 bp to 450 bp without abnormal sequence [27]. According to Figure 5B, it could be found that there were 557 ASVs in all groups, which were comprised of core intestinal microbiota in mice. There were 162 ASVs overlapped between M and NC, whereas the number of ASVs overlapped between JY, JM, MIX, and NC were 241, 227, and 264, respectively, indicating that compared with the M group, the gut microbiota compositions of diet intervention groups were closer to that of NC group, and the advantage of MIX group was more significant. Alpha diversity index (Figure 5C–F) showed no significant differences among different groups (*p* > 0.05), but it also can be seen that those of the M group were all decreased, and the community richness and diversity of mice could be improved to a certain extent after the intervention, which was similar to Zhang’s results [28]. Beta diversity analysis (Figure 5G–I) showed that the community structure of each sample in the NC group with high similarity was different from that of other groups, but the opposite trend was observed for M group with more dispersed samples. This trend could be ameliorated by *L. paracasei* JY062 and *L. gasseri* JM1, especially their combination.

**Figure 6:**
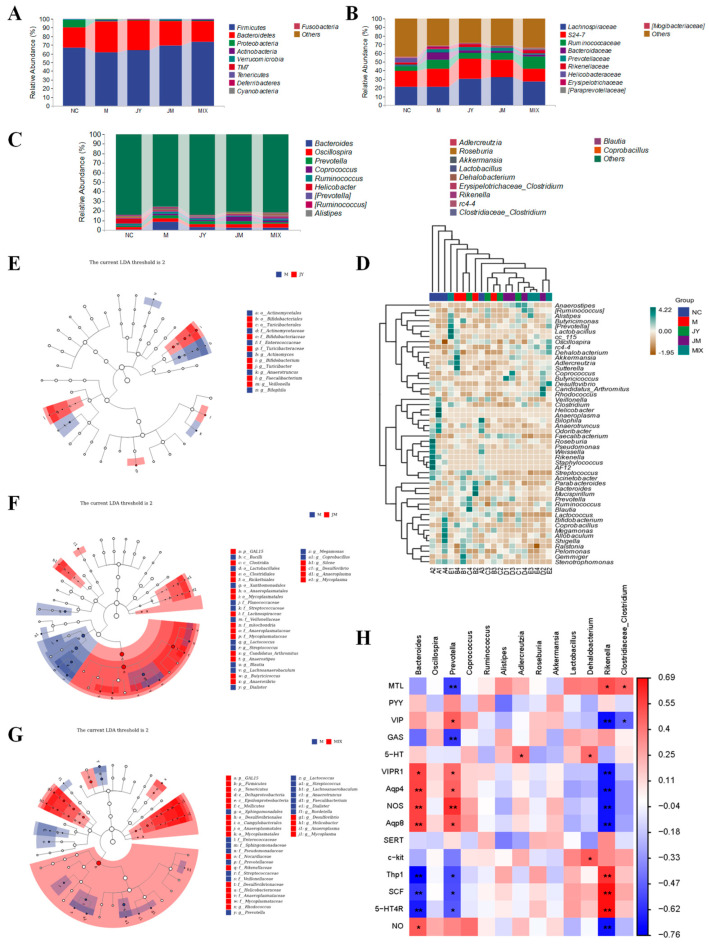
Differences in gut microbiota composition among the five groups (*n* = 4): (**A**) microbial composition at the phylum level; (**B**) microbial composition at the family level; (**C**) microbial composition at the genus level; (**D**) heatmap of genus composition combined with cluster analysis at genus level; (**E**) LDA Effect Size (LEfSe) analysis between M and JY groups; (**F**) LEfSe analysis between M and JM groups; (**G**) LEfSe analysis between M and MIX groups; (**H**) the correlation heatmap between gut microbiota and other physiological indexes. * *p* < 0.05 and ** *p* < 0.01 represent significant correlation between gut microbiota and SCFAs. (**A**) stands for the normal control group (NC), (**B**) for the model group (M), (**C**) for the *L. paracasei* JY062 group (JY), (**D**) for the *L. gasseri* JM1 group (JM), and (**E**) for the combination group (MIX).

As shown in Figure 6A, gut microbiota of mice in different groups was analyzed at the phylum level. Firmicutes and Bacteroidetes were predominant, accounting for about 90% of the total. The abundance of Firmicutes was decreased and Bacteroidetes was increased in the M group, whereas these trends were reversed in the probiotic groups, and notably the abundance of Firmicutes in the MIX group (73.74%) was higher than the N group (66.99%). At the family level (Figure 6B), the abundance of *Bacteroidaceae*, *Prevotellaceae*, and *Erysipelotrichaceae* in the M group was significantly increased, whereas that of *Rikenellaceae* was decreased. In addition to the reversal of their abundance after the intervention, *Lachnospiraceae* and *Ruminococcaceae* were increased simultaneously, especially in the MIX group. At the genus level (Figure 6C), compared with NC group, the abundance of *Bacteroides* and *Prevotella* was increased in the M group (8.63% and 3.08%) and decreased after the probiotic intervention, with the most significant changes in the MIX group (2.64% and 0.90%), which closed to the NC group (0.57% and 0.85%). It was noteworthy that the abundance of *Lactobacillus* in the MIX group (0.72%) was the highest among all groups, which was much higher than that in the NC group (0.29%). Simultaneously, the abundance of *Clostridiaceae_Clostridium* in the NC, M, JY, JM, and MIX groups were 0.23%, 0.11%, 0.08%, 0.13%, and 0.20%, respectively. It could be seen that the abundance of *Clostridiaceae_Clostridium* in the MIX group was closer to that in the NC group. As shown in Figure 6D, the samples in the NC group were aggregated, indicating that the community structures of gut microbiota were highly similar, whereas the gut microbiota composition in the M group was changed with certain differences. After the intervention, the samples of the JY, JM, and MIX groups were relatively clustered and had a high similarity, which further supported the results of Beta diversity analysis. According to the LDA Effect Size (LEfSe) analysis (Figure 6E–G), compared with the M group, *Bifidobacterium*, *Faecalibacterium*, and *Veillonella* were mainly enriched in the JY group. *Clostridiales*, *Lachnospiraceae*, *Butyricicoccus,* and *Anaerovbrio* were mainly enriched in the JM group. *Rikenellaceae* were mainly enriched in the MIX group. The whole was consistent with the above description. Simultaneously, there was a certain correlation between gut microbiota and other physiological indexes (Figure 6H), the mRNA expression of VIPR1, Aap4, Aap8, and NOS were positively correlated with *Bacteroides* and *Prevotella* and negatively correlated with *Rikenella*, whereas the mRNA expression of Thp1, SCF, and 5-HT_4_R showed an opposite correlation with the above bacteria genus (*p* < 0.05, *p* < 0.01). MTL was positively correlated with *Rikenella* and *Clostridiaceae_Clostridium* but negatively correlated with *Prevotella,* whereas VIP showed an opposite trend to MTL in terms of correlation with the above bacteria.

### 3.6. Effects on SCFAs of Mice with GI Motility Disorder

**Figure 7:**
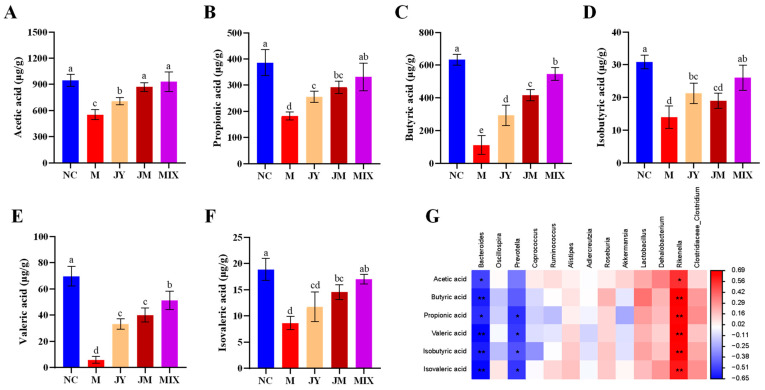
The effect of probiotics on short-chain fatty acids (SCFAs) in mice: (**A**) content of acetic acid; (**B**) content of propionic acid; (**C**) content of butyric acid; (**D**) content of isobutyric acid; (**E**) content of valeric acid; (**F**) content of isovaleric acid; (**G**) correlation between gut microbiota and SCFAs. Different letters (a, b, c, d and e) denote significant differences between different groups of mice (*p* < 0.05). * *p* < 0.05 and ** *p* < 0.01 represent significant correlation between gut microbiota and SCFAs. NC stands for the normal control group, M for the model group, JY for the *L. paracasei* JY062 group, JM for the *L. gasseri* JM1 group, and MIX for the combination group.

Current studies have shown that the levels of SCFAs could affect the intestinal motility of mice, and some probiotics have been shown to regulate the production of intestinal SCFAs [29,30]. The contents of SCFAs (acetic acid, propionic acid, butyric acid, isobutyric acid, valeric acid, and isovaleric acid) in the M group were significantly decreased (*p* < 0.05), whereas those were improved to different extents after the intervention, especially in the MIX group; additionally, there were differences between the two strains, and the improvement effect of the *L. gasseri* JM1 intervention was higher than that of the *L. paracasei* JY062 intervention (Figure 7A–F). Further, the correlation analysis between SCFAs and differential bacterial genus (Figure 7G) showed that the contents of the above SCFAs were positively correlated with *Rikenella* (*p* < 0.05, *p* < 0.01) but negatively correlated with *Bacteroides* (*p* < 0.05, *p* < 0.01). Among them, propionic acid, isobutyric acid, valeric acid, and isovaleric acid were also negatively correlated with *Prevotella* (*p* < 0.05). In addition, all the above SCFAs showed a certain positive correlation with *Lactobacillus*, *Dehalobacterium*, and *Clostridiaceae_Clostridium*. Simultaneously, we found that the abundance of *Lactobacillus*, *Dehalobacterium*, *Clostridiaceae_Clostridium*, and *Rikenella* were increased, whereas those of *Bacteroides* and *Prevotella* were decreased after the intervention. The probiotic combination could improve the contents of SCFAs by regulating gut microbiota.

## 4. Discussion

At present, many studies have proven the probiotic function of lactic acid bacteria and have been applied in functional food [31,32]. Herein, the mechanism by which *L. paracasei* JY062 and *L. gasseri* JM1 and their combination alleviated GI motility disorders was explored, including the regulation of GI regulatory peptides, the balance of gut microbiota, and the regulation of SCFAs.

Loperamide is a μ opioid receptor agonist that acts on intestinal smooth muscle to inhibit the contraction of intestinal smooth muscle and suppress intestinal motility, thereby prolonging the residence time of food in the small intestine [33]. Blockade of μ opioid receptors and antagonism of GI calmodulin were the molecular mechanisms by which loperamide affected intestinal motility [21]. In rodent models, it has been used to cause delayed gastric emptying and decreased intestinal motility [16]. In the present study, with 5 mg/(kg·d) body weight of loperamide for 7 days [34], the mice showed a significant decrease in gastric emptying rate and small intestinal propulsive rate, as well as symptoms of defecation difficulty and dark stool, indicating successful modeling. The movement of GI contents is primarily driven by GI motility, which can be detected by measuring gastric emptying rate and small intestine propulsive rate. After the probiotic intervention, those were significantly increased in mice induced by loperamide, which was consistent with Tang’s results [15]. Moreover, when feces remain in the intestine for a longer time, more water is absorbed, resulting in a decrease in fecal moisture content that is detrimental to intestinal motility. Aquaporins in the cell membrane can selectively transport water molecules [35]. The decreased transcription level of Aqp4 and Aqp8 improved the absorption of water in the intestinal lumen after the intervention. Simultaneously, fecal moisture content showed an increasing trend. These initial data suggested that probiotics may improve GI motility.

Moreover, the HE pathological analysis showed that the probiotic combination could improve the organismal injury induced by loperamide. Of note was the appearance of Paneth cells in the small intestine, which secreted some antimicrobial molecules such as defensins into the villi of the intestine lumen to help maintain the GI barrier when bacteria or bacterial antigens invade the organism [36]. Unexpectedly, the appearance of Paneth cells in the M group may be related to the stress response made by innate autoimmunity [37].

GI regulatory peptides, including excitatory regulators (MTL and GAS) and inhibitory regulators (PYY and VIP), play an essential role in the regulation of GI motility. MTL could cause intense phase III contraction of interdigestive migrating contractions, which are closely associated with gastric emptying and intestinal propulsion [38]. GAS is the peptide hormone secreted by G cells that strongly stimulates the secretion of gastric acid and pepsin [39]. In our study, the levels of MTL and GAS were both increased, and these were consistent with previous studies [14]. PYY is a typical appetite-suppressing hormone secreted by L cells, which could inhibit gastric acid secretion and GI motility, causing the body to feel satiety and reduce food intake [40]. We found that PYY was increased in mice induced by loperamide, whereas it was improved after the probiotic intervention. Some studies have demonstrated that VIP could relax smooth muscle and slow down small intestinal motility [41]. The majority of VIP actions were mediated by the expression of VIPR1 on the epithelial cells, cholinergic excitatory motor neurons innervating longitudinal muscles, cholinergic secretomotor neurons, and mucosal mast cells [42]. However, the downregulated transcription of VIPR1 after probiotic intervention in this study was consistent with Ren’s results [19], whereas the improvement to VIP was not significant, probably related to the failure of strain intervention to improve the expression of VIPR1 protein. Meanwhile, it has been demonstrated that endogenous VIP was released by numerous stimuli of at least two VIP-positive nerve populations: cholinergic and non-cholinergic VIP-releasing nerves [25]. This may also be one of the influencing factors in our study, but further research is needed. Therefore, *L. paracasei* JY062 and *L. gasseri* JM1 could promote GI motility by increasing excitatory regulators (MTL and GAS) and decreasing inhibitory regulators (PYY).

Neurotransmitters, including the excitatory factor 5-HT and the inhibitory factor NO, are crucial in GI regulation. 5-HT is mainly synthesized by enterochromaffin cells (EC) in the presence of tryptophan hydroxylase 1 (Tph1) and released into the lamina propria to act by activating the corresponding 5-HT receptor (5-HTR). Among the seven families of 5-HTR, G protein-coupled receptor 5-HT_4_R is the most in contact with GI contents, which is mainly responsible for the regulation of intestinal secretion [43,44]. Simultaneously, it will be inactivated under the action of the serotonin transporter (SERT), and the reaction is terminated [45]. Tph1 and 5-HT_4_R with upregulated transcription and SERT with downregulated transcription after probiotic intervention promoted the binding of 5-HT to 5-HT_4_R and improved intestinal motility better, which was consistent with previous results [46]. Surprisingly, it has also been shown that it can promote the secretion of GI hormones such as MTL [6], and the improvement of GI hormones may be related to 5-HT in our study. On the contrary, NO was considered as the main non-adrenergic, non-cholinergic inhibitory neurotransmitter responsible for smooth muscle relaxation [47]. Studies have shown that VIP could stimulate the production of NO [48], but there was no significant improvement in VIP after the probiotic intervention, indicating NO may still be affected in other mechanisms. Notably, nitric oxide synthase (NOS) could catalyze the synthesis of NO [49]. We found that the mRNA level of NOS was decreased after the probiotic intervention, especially the probiotic combination, which showed the same trend as the concentration of NO. Therefore, the probiotic combination could improve GI motility disorder by NO by regulating the transcription level of NOS.

ICC, important pacemaker cells in the intestine, are responsible for the generation of slow wave potential and smooth muscle contraction [50]. The dimer was formed by the combination of stem cell factor (SCF) and tyrosine kinase receptor c-kit that specifically labeled ICC, which regulated the proliferation and differentiation of ICC [51]. If this pathway was blocked or inhibited, resulting in a decrease in the number of ICC [52]. We found that mRNA expression of c-kit and SCF and protein expression of c-kit and SCF tended to increase after the probiotic intervention, indicating that *L. paracasei* JY062 and *L. gasseri* JM1 could increase ICC numbers by stimulating SCF/c-kit pathway.

The abundance and diversity of gut microbiota are crucial in GI diseases. In our study, we explored the effects of loperamide, single probiotics, and the combination of *L. paracasei* JY062 and *L. gasseri* JM1 on gut microbiota of mice with GI motility disorder. Specifically, the abundance of *Bacteroides*, *Prevotella*, and *Erysipelotrichaceae* was increased in the M group and significantly reduced after the probiotic intervention. It was generally accepted that gram-negative Bacteroidetes contained two dominant genera (*Bacteroides* and *Prevotella*) that had the potential to be linked with chronic inflammatory conditions [53], which may be one of the causes of inflammatory cell infiltration in the M group known by HE pathological analysis. Previous studies found that *Erysipelotrichaceae* was observed in diet-induced obese animals [54,55], and the increased abundance of *Erysipelotrichaceae* in M group was indicated that there may be a risk of lipid metabolism disorders in loperamide-induced GI motility disorder. It could be seen that the intestine of mice in the M group was more likely to be colonized by harmful bacteria, thus disrupting the balance of the intestinal microenvironment and potentially increasing the risk of other diseases. Conversely, the abundance of *Lachnospiraceae*, *Ruminococcaceae*, and *Rikenellaceae* was improved under probiotic intervention. Among them, *Lachnospiraceae* and *Ruminococcaceae* belonging to the Firmicutes phylum are other important bacteria for producing SCFAs and might be related to improving cardiovascular health [56,57]. *Rikenellaceae* belonging to the Bacteroidetes phylum could produce acetic acid and propionic acid and have the potential to protect against cardiovascular and metabolic diseases associated with visceral fat [58,59]. Interestingly, we found a significant increase in the abundance of *Rikenellaceae*, despite a decrease in that of Bacteroidetes. It is widely recognized that *Lactobacillus* and *Bifidobacterium* could regulate intestinal metabolites, reduce inflammation, and improve immunity [13,60]. Additionally, it was important that members of *Clostridiaceae* are known for the production of butyrate by carbohydrate fermentation [61]. Likewise, the increase of *Lactobacillus* and *Clostridiaceae_Clostridium* in the MIX group and *Bifidobacterium* in the JY group were shown, which was similar to previous results [11,62]. Our results suggested that the single and combination of *L. paracasei* JY062 and *L. gasseri* JM1 improved the gut microbiome of mice, where the improvement of multiple GI functional parameters was related to the changes in microbiome composition by correlation analysis. Thus, the changes in host GI function could be reflected by changing gut microbiota composition triggered by the ingestion of probiotics.

Furthermore, the interaction between gut microbiota and SCFAs could be demonstrated by the fact that SCFAs are important metabolites of microbiota metabolism, and they can inhibit the growth of harmful bacteria and stimulate the growth of beneficial bacteria [63,64]. SCFAs showed an indispensable role in intestinal health, for instance, acetic acid could promote intestinal motility by promoting the absorption of water and electrolytes, and propionic acid could combine with cations to form propionate to the liver to participate in gluconeogenesis to provide energy for extensive metabolism and intestinal motility [65]. Apart from that, SCFAs have more functions, including the anti-inflammatory effect of butyric acid and the inhibitory effect of valeric acid on liver cancer [66,67]. Although there was no significant improvement in some SCFAs (isobutyric acid and isovaleric acid) after the intervention of single *L. gasseri* JM1 and *L. paracasei* JY062 respectively, SCFAs were mostly increased after the intervention of their combination, along with an increase in SCFAs-producing bacteria, further indicating their interaction. Therefore, the improvement in GI motility could be influenced by the increased SCFAs due to the change in gut microbiota.

## 5. Conclusions

In summary, the probiotic combination of *L. paracasei* JY062 and *L. gasseri* JM1 could alleviate GI motility disorder in mice, specifically by improving the secretion of MTL, GAS, PYY, 5-HT, and NO, and increasing the expression of c-kit and SCF protein. In addition, gut microbiota was regulated through an increase in SCAFs-producing bacteria (*Lactobacillus*, *Rikenellaceae*, and *Clostridiaceae _Clostridium*) and a decrease in harmful bacteria (*Bacteroides* and *Prevotella*). Additionally, the concentrations of acetic acid, propionic acid, butyric acid, and valeric acid were also increased mostly. Thus, it showed that the combination of *L. paracasei* JY062 and *L. gasseri* JM1 could balance intestinal homeostasis through multiple actions to alleviate GI motility disorder in mice induced by loperamide. This study could provide a potential adjuvant therapeutic option for alleviating GI motility disorders and provide strains for the subsequent development of functional foods.

## Figures and Tables

**Table 1:** The specific RT-qPCR primers for target genes.

Gene	Primer Sequences (5′→3′)	
	Forward	Reverse
GAPDH	TGACCTCAACTACATGGTCTACA	CTTCCCATTCTCGGCCTTG
Aqp4	CAGCATCGCTAAGTCCGTCTTCTAC	ACCGTGGTGACTCCCAATCCTC
Aqp8	GGAACATCAGCGGTGGACACTTC	GGGAATTAGCAGCATGGTCTTGAGG
Thp1	ATCCGTCCTGTGGCTGGTTACC	AGGTGTCTGGCTCTGGAGTGTAG
5-HT_4_R	AAGGCTGGAACAACATCGGCATAG	ACCACAGAGCAGGTGATAGCATAGG
SERT	CGTCGTCGTGTCTTGGTTCTATGG	GAACAGGAGAAACAGAGGGCTGATG
c-kit	GATCTGCTCTGCGTCCTGTTGG	AACTCTGATTGTGCTGGATGGATGG
SCF	TGCGGGAATCCTGTGACTGATAATG	CCGGCGACATAGTTGAGGGTTATC
VIPR1	AAGAAGGCTGGTCACAACTGGAAC	AGAACTCAGTCTGTTGCTGCTCATC
NOS	GTCAGAAGATGTCCGCACCAAGG	TGTTCACCTCCTCCAGCCTGTC

## Data Availability

Data in the project are still being collected, but all data used in the study is available by contacting the authors.
